# A Computational and Experimental Investigation of the Phonon and Optical Properties of Au_2_P_3_

**DOI:** 10.3390/ma12040555

**Published:** 2019-02-13

**Authors:** Michael Snure, Timothy Prusnick, Elisabeth Bianco, Stefan C. Badescu

**Affiliations:** 1 Air Force Research Laboratory, Sensor Directorate, 2241 Avionics Circle, Wright Patterson AFB, OH 45433, USA; timithoy.prusnick.ctr@us.af.mil (T.P.); catalin.badescu.1@us.af.mil (S.C.B.); 2 KBRWyle Laboratories, 2241 Avionics Circle, Wright Patterson AFB, OH 45433, USA; 3 Air Force Research Laboratory, Materials and Manufacturing Directorate, 2941 Hobson Way, Wright Patterson AFB, OH 45433, USA; elisabeth.bianco.ctr@us.af.mil

**Keywords:** gold phosphide, Raman spectroscopy, density functional theory, band structure, dielectric function

## Abstract

In a combined experimental and theoretical study of gold phosphide (Au_2_P_3_), we investigate its vibrational properties, band structure, and dielectric properties, providing new insight into the properties of this underexplored material. Using a simple synthesis route, Au_2_P_3_ thin films were produced, enabling the first reported Raman analysis of this material. Coupled with first-principles calculations of these Raman modes, this analysis reveals that low-frequency vibrations are due to Au or mixed Au to P, and at higher frequencies, they are due to P vibrations. Further band structure and dielectric calculations reveal Au_2_P_3_ to be a narrow band (0.16 eV) indirect semiconductor. This work helps to fill major gaps in our understanding of key properties in this material that will benefit future research in this field.

## 1. Introduction

Metal phosphides are of interest for a number of applications for electronics and semiconductors, catalysts, hydrogen storage, and magnetic devices. Among this broad and diverse materials group, gold phosphide (Au_2_P_3_) has remained largely unstudied. This is likely due, in part, to its instability [1] decomposing to Au and P_4_ at one atm at temperatures above 700 °C [2]. To this point, Au_2_P_3_ has been the most widely studied in the formation of Ohmic contacts for InP, which forms upon annealing Au on InP [3,4]. Transmission electron microscopy investigations show that rough polycrystalline Au_2_P_3_ layers form at the InP surface [3]. The formation of Au_2_P_3_ between Au and InP is responsible for a significant drop in the contact resistance for both n and p-type InP [5]. Additionally, nanostructured Au_2_P_3_ has recently been synthesized by solution methods forming networks of nanoparticles (~5 nm) [6,7], which were demonstrated to be efficient catalysts for hydrogen [6]. As such, this limited line of investigation leaves a great deal unknown about this material.

Early studies on bulk polycrystalline gold phosphides reported the synthesis of multiple phases and compositions, which later were found to contain additional elements and contaminates. Through the direct reaction of elemental Au and red phosphorus powders, phase pure Au_2_P_3_ was formed and identified as the only stable gold phosphide phase [8]. Due to the highly reactive nature of P and the stability of ternary gold phosphides, synthesis methods using transport agents and mineralizers can result in ternary compounds such as Au_3_SnP_7_ and Au_7_IP_10_ [8,9], complicating synthesis. From crystalline samples, Jeitschko and Moller [8] described a detailed structural analysis of monoclinic Au_2_P_3_, providing complete powder diffraction data and structural description. Beyond this, little is known about its optical, conduction, or phonon properties. As such, calculations of band structure and phonon dispersion would be beneficial for Raman, IR, and other optical characterization, as well as examining its usefulness for the above applications. In this paper, we first describe the experimental methods for the synthesis, structure, and Raman characterization, and then coupled these experimental results with first-principles phonon and band structure calculations to fill gaps in the general knowledge of this material.

## 2. Materials and Methods

Gold phosphide films were formed on Au foil using a horizontal hot wall chemical vapor deposition (CVD) reactor with a three-zone furnace. Phosphine was used at the P source, and H_2_ was used as the carrier gas. A reactor pressure of 700 Torr and furnace profile of 650/450/350 °C (Z1/Z2/Z3) across the three zones was used. Substrates were loaded in between Z1 and Z3 to achieve temperatures between 600 and 300 °C. Once temperatures were reached and stabilized, PH_3_ was introduced at a flow rate of 80 sccm with 160 sccm H_2_ flow. After three hours, the reactor was cooled to 200 °C, and the PH_3_ was turned off. Samples were unloaded and stored in a N_2_ glove box with <0.1 ppm O_2_ and H_2_O to prevent oxidation. 

As-deposited films were characterized using optical microscopy, X-ray diffraction (XRD), transmission electron microscopy (TEM), energy-dispersive X-ray spectroscopy (EDS), and Raman spectroscopy. X-ray diffraction using an Empyrean X’pert Pro system with a four-bounce Ge monochrometer was used for structural characterization and phase identification. The TEM-ready samples were prepared using the in situ focused ion beam (FIB) lift-out technique, and high resolution (HR)TEM images were acquired on an FEI Titan Themis TEM equipped with a post-specimen aberration corrector (Cs=0). An accelerating voltage of 300 kV was used in Free Control mode with the C3 lens off and a BM-UltraScan 2048 x 2048 CCD detector. Scanning tunneling electron microscopy (STEM) images and EDS were acquired on an FEI Talos TEM operated in STEM mode at 200 kV using a high-angle annular dark field (HAADF) detector and an FEI SuperX quad-core EDS detector, respectively. Raman measurements were carried out under a N_2_ atmosphere in a Linkam stage using a Renishaw inVia system. An accumulation of 20 scans, each of 30-s duration, was collected using a 250-µW 514 nm excitation source, 20-µm slits, and a 3000 line/mm grating for each measurement.

First-principles calculations based on density functional theory (DFT) were performed using the Vienna AB-Initio Simulation Program (VASP) [10]. We used the projector-augmented wave function (PAW) pseudopotentials, with an energy cutoff E_Cut_ = 255 eV [11]. The Brillouin zone was sampled with uniform 6 × 6 × 8 k-point mesh. Structural relaxation and phonon mode calculations were performed at the generalized gradient approximation level (GGA) using the Perdew–Burke–Ernzerhof functional [12]. The electronic band structure and the density of states were obtained with the Heyd–Scuseria–Ernzerhof (HSE06) functional [13], and then adjusted with self-consistent quasiparticle scGW0 correction [10,14]. The first Brillouin zone and the symmetry points were obtained using the AFLOW software [15] based on reference [16].

## 3. Results and Discussion

**Figure 1:**
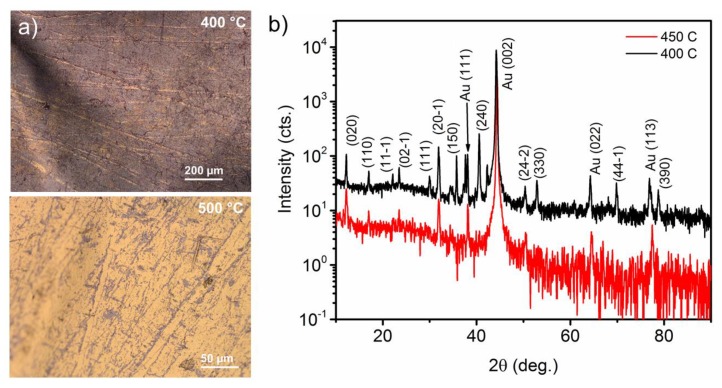
Structural characterization of Au_2_P_3_ on Au films (**a**) optical images of films grown at 400 °C and 500 °C, and (**b**) X-ray diffraction (XRD) spectra from films grown at 400 °C and 450 °C. All of the peaks have been all indexed to Au_2_P_3_ (JCPDS# 98-000-8058) or Au (JCPDS# 98-004-4362). Unlabeled peaks can be assigned to Au_2_P_3_.

Gold phosphide films on Au were synthesized using a simple process of reacting PH_3_ with Au in a CVD system. The process is fairly robust, with Au_2_P_3_ forming over a wide range of pressures between 20–700 Torr and temperatures between 400–500 °C. Over these ranges, parameters were found to have a significant impact on coverage (Figure 1). Below 400 °C, red phosphorus films form, and much above 500 °C, no growth was observed. Due to the metastable nature of Au_2_P_3_, we investigated the annealing of films in a N_2_ or H_2_ ambient at 700 Torr over a temperature range of 500 °C to 650 °C. Films were found to completely disappear at temperatures >600 °C, where no sign of Au_2_P_3_ was observable in optical, scanning electron miscopy, or XRD measurements. This range of decomposition temperatures is consistent with the thermodynamic investigations by Myers et al. [17] and considerably higher than those reported for the Au_2_P_3_–InP system [18]. To identify the phase and investigate the structure of the films, XRD (Figure 1a) was performed on films grown at 400 °C and 

450 °C. The diffraction pattern from both films shows peaks corresponding to Au_2_P_3_ (JCPDS# 98-000-8058) and Au. Due to the higher coverage area of the 400 °C sample, we see a much more detailed diffraction spectra for the Au_2_P_3_ film, which is in excellent agreement with previous reports for poly and nanocrystalline samples [6,7,8]. From the spectra, four peaks were assigned to the Au substrate, which is predominantly (002) oriented, with all of the other peaks assignable to the monoclinic Au_2_P_3_ structure. According to XRD, films can be identified as polycrystalline with no clear preferred orientation, in spite of the oriented substrate. Temperature, within this narrow range, does not appear to have an effect on the Au_2_P_3_ film orientation.

**Figure 2:**
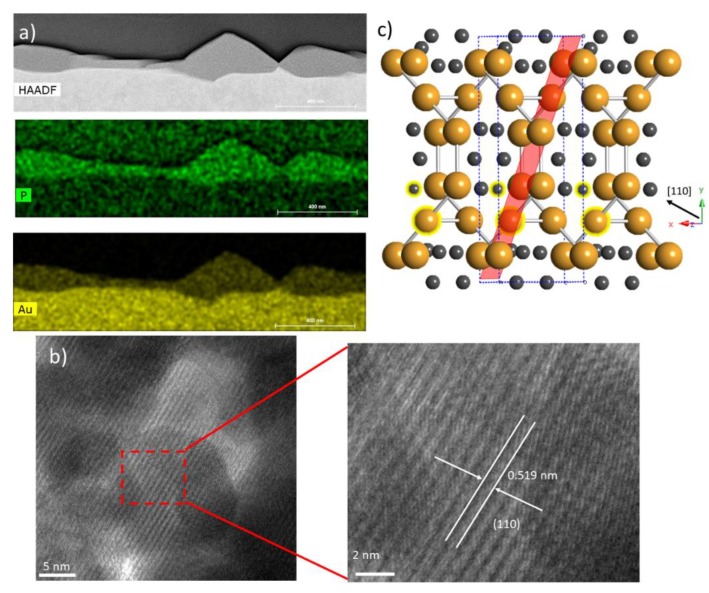
(**a**) High-angle annular dark field (HAADF) image of film cross-section (top) and energy-dispersive X-ray spectroscopy (EDS) chemical maps of P (middle) and Au (bottom); (**b**) High-resolution TEM of film and measured layer spacing; (**c**) Schematic of the Au_2_P_3_ monoclinic crystal structure where the orange atoms are Au and the black atoms are P. The red plane corresponds to the (110).

Closer examination of the crystal structure and chemical make-up of these films was performed using cross-sectional TEM, STEM, and EDS, as shown in Figure 2a,b. HAADF images of the cross-section clearly show the formation of films on the surface of the Au foil (Figure 2a). The film thickness is clearly non-uniform, with significant height variation ranging from 50 nm to 200 nm. This morphology is quite similar to those reported on InP [4]. Elemental EDX maps of Au and P over this same region delineates the substrate from the film matching the HAADF images. These images show a uniform distribution of P and Au throughout the film, and a low P background in the Au substrate. The high-resolution TEM images show the films to be crystalline and reveal a layered structure that is consistent with previous reports on Au_2_P_3_ nanostructures [6]. The measured spacing between these layers is ~0.519 nm, which is matched with the calculated d-spacing for (110). Figure 2c shows a schematic of the crystal structure. We can see the clear layered nature of this structure along the [110] formed by planes of Au, which is consistent with our TEM observations. 

The combined structural and elemental analysis confirm the formation of thin film monoclinic Au_2_P_3_, which we now use for Raman characterization. Figure 2a shows the room temperature Raman spectra from Au_2_P_3_ films grown at 400 °C and 450 °C. Eleven Raman peaks are observed over the range of 50 to 1000 cm^−1^, as listed in Table 1. Raman spectroscopy is an important technique for identifying materials, and it is quite prevalent in the study of nanomaterials. It is particularly useful for very small sample sizes or mapping across structures where traditional bulk characterization methods (XRD) may not be suitable. However, to this point, we are unaware of any previous reports on the Raman spectra of Au_2_P_3_. Coupled with the structural analysis provided in figures 1 and 2, we have confidence that the presented Raman spectra are characteristic of the monoclinic phase of Au_2_P_3_. To both strengthen our confidence as well as gain a deeper understanding of the structural, vibrational, and electronic properties, we performed DFT using VASP. The crystallographic cell contains 20 atoms, and the relaxed crystal assumes a c-centered monoclinic structure of type MCLC_3_ [16] with calculated lattice parameters a=14.56, b=4.75, c=5.91 Angstrom, and *β* = 109°.

**Figure 3:**
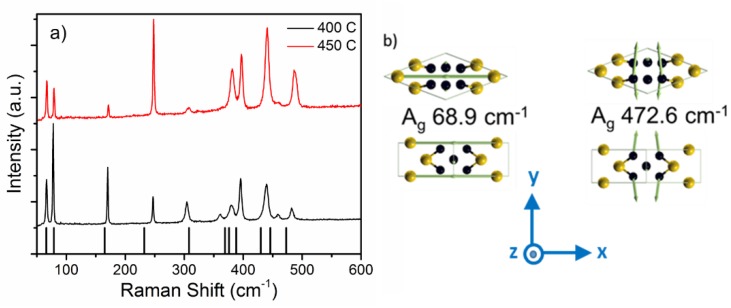
(**a**) Raman spectra from Au_2_P_3_ film on Au deposited at 400 °C, 450 °C, and calculated Raman modes. (**b**) Schematics of atomic vibrations, for example low-frequency and high-frequency Raman modes; the inset shows the Cartesian axis orientation, with axis *a* parallel to x and axis c parallel to y.

The primitive cell (shown in Figure 3b and used in the DFT calculations) contains 10 atoms. The calculated vibrational modes and measured Raman modes are presented in Table 1 and Figure 3a. According to calculations, there are 12 Raman active modes and 15 IR modes. The 11 Raman modes from 68.9 to 472.6 cm^−1^ are well matched with the experimentally observed values. Only the B_g_ mode at 41 cm^−1^ could not be observed due to the limit of the Rayleigh filtering. Due to the Au substrate, IR spectra were not able to be measured, and thus require further work to experimentally verify. However, these results provide a database for its future characterization and identification, which are important for the further study of this intriguing material.

Calculations of the Au_2_P_3_ vibrational modes provides more insight into this material than just mode identification. Evaluation of the atomic displacement that is associated with each mode reveals a clear physical distinction between the low and high-frequency modes. At frequencies above 158.6 cm^−1^, the vibrations are due to P–P bonds, while at lower frequencies, they are due to Au or mixed Au and P vibrations, as shown in Figure 3b. This may be a consequence of two intercalated sublattices—of phosphorous and gold atoms—with the former more strongly bonded to one another. This finding is consistent with the bond calculation presented by Xu et al. [19] that suggests much stronger covalent bonding between P atoms than Au atoms. Knowing the atomic origin of these modes may enable us to predict or interpret changes in the structure. If we compare the Raman spectra in Figure 3a, we observe the significant reduction in the relative intensity of modes at 67 cm^−1^ and 78 cm^−1^ for the sample grown at 450 °C, which are dominated by the vibration of Au atoms. This may suggest a tendency toward phase separation in the higher-temperature samples.

**Figure 4:**
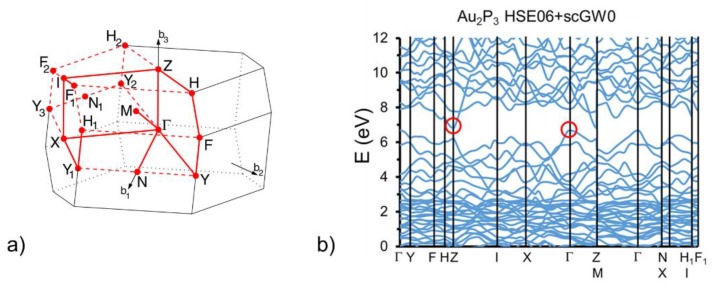
(**a**) The first Brillouin zone of Au2P3 obtained with the AFLOW [15,16]; (**b**) Electronic band structure of Au2P3 along the k-point path in (**a**). The red circles mark the top of the valence band (at the Γ point) and the bottom of the conduction band (at Z point), with a band gap of 0.160 eV.

In recent articles, it has been suggested that Au_2_P_3_ is expected to have interesting optical properties; however, not much is really known about these properties [7,19]. Reference [8] described Au_2_P_3_ as having metallic conduction and focuses on electron, interatomic interactions, and phase transitions. Reference [19] discussed the finite band gaps and the resulting optical properties of small five-atom Au_2_P_3_ clusters. By virtue of their molecular size, the latter have band gaps of the order of eV, and cannot be used to interpret the band gaps and optical properties of extended systems, where periodic boundary condition calculations are in order. As such, the work presented here is an important addition to the study of Au_2_P_3_. The metallic conductivity in [8] can be interpreted either as a lack of a band gap or as native doping in the presence of a very small band gap. Our calculated electronic band structure is given in Figure 4, and to the best of our knowledge, it is the first for bulk Au_2_P_3_ in the literature. The standard DFT method describing a proper crystal structure had to be augmented in order to obtain an accurate electronic structure. The latter is obtained using the HSE06 hybrid functional HSE06 [13] and self-consistent quasiparticle (scGW0) corrections [10,14]. Convergence was obtained within one meV in 10 iteration steps. The result is that Au_2_P_3_ is an indirect band gap semiconductor with a transition from Γ to Z and a zone-center band gap of 0.160 eV. The valence and the bottom of the conduction band result from the overlap between the partially filled s1d10 orbitals of Au atoms and the partially filled s^2^p^3^ orbitals of P atoms.

**Figure 5:**
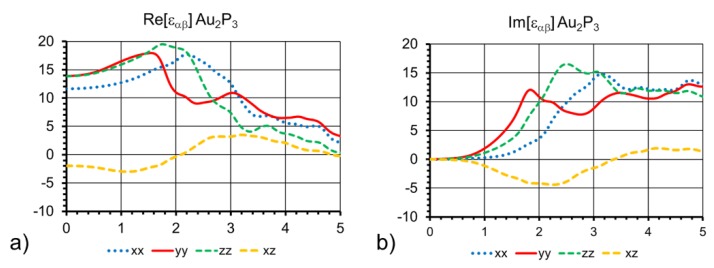
(**a**) Real and imaginary (**b**) parts of the dielectric function from HSE06+scGW0 ab-initio calculations. The three diagonal components and the off-diagonal component in the (a,b)-plane are shown.

The free-carrier contribution to the real and imaginary parts of the dielectric function is shown in Figure 5. This is due to valence to conduction-band excitation, and covers a large range of energies. This type of data can be compared to ellipsometry measurements and optical conductivity data to extract the characteristic scattering times for the quasi-free electron Drude model. Using the orientation given in Figure 3b, the three types of P atoms in the primitive cell give rise to three branches at the top of the valence band with different p_x, p_y, and p_z orbital characters. This results in different optical transition elements to the conduction band and the anisotropy of the dielectric tensor. Using our current process, we only have access to thin layers of phosphide with high roughness on a relatively thick Au substrate, making experimental verification of the dielectric function via transmission reflectance and ellipsometry unfeasible. A comparison of the computed dielectric function with experiments will become possible when standalone bulk or thin films on an insulator become available to the community.

As noted above, the band gap of the bulk material is much smaller than the separation between the occupied and unoccupied orbitals of small clusters [19]. The small band gap of 160 meV found here is only somewhat larger than the energies of the bulk phonon modes from the first section, which have a maximum at ~50 meV. This means that an experimentally measured dielectric function at room temperature would present a significant overlap at low energies between the valence–conduction band transitions shown in Figure 4b, and an IR signal from the phonon modes. In addition, if this material is natively n-doped (as suggested by its metallic conductivity), the dielectric function is expected to have a plasmonic component at low energies in the low-energy range comparable to the band gap. The topics of native point defect formation energies and plasmonic resonances are outside the scope of this work.

## 4. Conclusions

Experimental measurements supported by first-principles modeling reveal key details of the vibrational modes and band structure of Au_2_P_3_. Reacting phosphine with Au substrates, we present a simple process to form monoclinic Au_2_P_3_ thin films, which is unequivocally identified by X-ray diffraction and TEM structural analysis. Utilizing these films, Raman spectra are presented with excellent agreement between experiment and theory, providing, for the first time, information on the vibrational modes. The DFT calculations in this work further supply the electronic structure and optical properties of this material in bulk form. Au_2_P_3_ is found to be an indirect semiconductor with a 0.16-eV band gap. This information will be important to those working in the field of metal phosphides, providing important details on Raman and I.R. spectra, as well as answering questions about the electronic structure and optical properties.

## Figures and Tables

**Table 1:** Calculated vibrational modes and measured Raman modes for Au_2_P_3_. I.R. = infrared, N/O = not observed.

Mode	Calc. (cm^−1^)	Expt. (cm^−1^)	Mode	Calc. (cm^−1^)	Expt. (cm^−1^)
B_g_	41	N/O	B_g_	369.4	363
A_g_	68.9	67	A_g_	376.2	378
B_g_	78.9	78	A_g_	397.2	396
B_g_	165.4	171	A_g_	429.5	438
A_g_	231.8	247	B_g_	446.3	464
B_g_	307.7	305	A_g_	472.6	483
I.R. Mode Calc.	A_u_, B_u_, B_u_, B_u_, A_u_, A_u_ B_u_, B_u_, A_u_, B_u_, B_u_ A_u_, B_u_, A_u_, A_u_		56.8, 57.1, 65.6, 72.2, 78.4, 107.7, 136.2, 158.6, 191.7, 288.5, 382.9, 383.8, 407.1, 423.9, 469.4 cm^−1^		
